# Marbofloxacin Suppresses Breast Cancer Growth Through Oxidative Stress and Metabolic Reprogramming with Predicted HSP90AA1–EGFR Network Modulation

**DOI:** 10.3390/ph19091327

**Published:** 2026-08-22

**Authors:** Mervenur Yavuz, Firli R. P. Dewi, İlknur Keskin, Turan Demircan

**Affiliations:** 1 Molecular Biology and Genetics Program, Institute of Natural Sciences, Mugla Sitki Kocman University, Mugla 48000, Turkey; ymervenuryavuz@gmail.com; 2 Department of Biology, Faculty of Science and Technology, Universitas Airlangga, Surabaya 60115, Indonesia; firli.rahmah@fst.unair.ac.id; 3 Research Center for Stem Cells Development, Universitas Airlangga, Surabaya 60115, Indonesia; 4 Histology and Embryology Department, School of Medicine, İstanbul Medipol University, İstanbul 34810, Turkey; 5 Medical Biology Department, School of Medicine, İzmir Bakircay University, İzmir 35665, Turkey

**Keywords:** breast cancer, drug repurposing, marbofloxacin, fluoroquinolone antibiotics, metabolomics, network pharmacology, molecular docking, EGFR

## Abstract

**Background:** Drug repurposing represents an accelerated and cost-effective approach to discovering novel oncologic therapeutics. Here, we investigated the anticancer potential and underlying mechanisms of marbofloxacin (MBF), a veterinary fluoroquinolone (FQ), against breast cancer (BC) cells. **Methods:** The cellular impacts of MBF on cell viability, anchorage-dependent growth, tumorigenicity, migration, apoptosis, proliferation, senescence, and mitochondrial function were thoroughly characterized. To further elucidate its mechanistic activity, real-time qRT-PCR, untargeted LC-MS/MS-based metabolomics, network pharmacology, and molecular docking analysis were integrated. **Results:** MBF suppressed BC cell growth by inhibiting cellular proliferation and migration, disrupting mitochondrial membrane potential, and inducing ROS-mediated apoptosis and irreversible cellular senescence. These phenotypic impacts were accompanied by upregulation of tumor suppressors such as *CDKN1A* and *PUMA* and downregulation of oncogenes including *MKI67*, *BIRC5*, and *BCL-2*. Metabolomic analysis revealed broad suppression of biosynthesis-related metabolic pathways, characterized by the depletion of critical polyamines and nucleotide pathways. Network pharmacology and molecular docking analyses identified EGFR and HSP90AA1 as putative hub proteins potentially associated with the observed anticancer phenotype. **Conclusions:** These results provide initial evidence that MBF induces metabolic and molecular rewiring in BC, highlighting its promise as a repositionable therapeutic candidate.

## 1. Introduction

Breast cancer (BC) represents the most diagnosed malignancy in women worldwide and ranks as the second most prevalent cancer overall, accounting for approximately 11.6% of total global cancer cases [1]. Despite the advances in molecular diagnostics, screening programs, and therapeutic interventions, BC remains a major global health burden [2]. BC comprises a heterogenous group of tumors characterized by distinct molecular, histological, and clinical features, including estrogen receptor (ER)-positive and triple-negative breast cancer (TNBC) subtypes [3]. Current treatment regimens involve surgery, radiotherapy, chemotherapy, endocrine therapy, targeted agents, and, more recently, immunotherapy [4,5]. The inherent complexity and high heterogeneity of the disease significantly obstruct conventional therapeutic approaches, often leading to therapy resistance and metastatic progression [6,7]. The high mortality and recurrence rates necessitate the identification of novel therapeutic strategies and candidates that can effectively circumvent current clinical constraints.

Drug repurposing is a therapeutic strategy involving the use of existing drugs outside their original indications, which has been developed to accelerate anticancer drug discovery while reducing the time, cost, and risks associated with conventional drug discovery pipelines, which typically require more than a decade of research and substantial financial investment [8,9]. Repurposed drugs offer several advantages, including established pharmacokinetic profiles, reported safety characteristics, reduced developmental costs, and shorter timelines to clinical implementation [10]. The identification of non-oncologic drugs with understudied anticancer activities has gained significant attention considering these advantages.

We investigated the potential anticancer impacts of marbofloxacin (MBF), a third-generation synthetic fluoroquinolone (FQ) antibiotic conventionally used in veterinary medicine, in BC. Its broad-spectrum bacterial activity has been characterized mostly against Gram-negative bacteria, *Mycoplasma* species, and various Gram-positive pathogens and widely indicated for treatment of respiratory, gastrointestinal, skin, and urinary tract infections in several animal species, including cats, dogs, pigs, and horses [11,12,13,14]. Currently, it is commercially available under names such as Marbocyl, Zeniquin, and Forcyl, and favored for its high bioavailability and tissue penetration [15]. In dogs and pigs, MBF exhibits nearly 100% oral bioavailability [12,13], while in cats, an 8–10 h half-life with significant excretion via urine was reported previously [16]. Moreover, the drug shows approximately 98% bioavailability when administered subcutaneously, with a 0.25 L/kg/h clearance rate in horses [11]. The literature indicates that MBF is generally well tolerated, with limited systemic toxicity; however, subacute studies have established a no-observed-adverse-effect level of 4 mg/kg/day [13,15]. These established safety profiles, particularly MBF’s ability to reach high concentrations in target tissue, provide a strong clinical rationale for exploring its potential in oncology.

The present study aimed to evaluate MBF’s anti-neoplastic potential in ER-positive (MCF-7) and TNBC (MDA-MB-231) cells. To the best of our knowledge, this is the first study systematically exploring its activity on BC cells through comprehensive phenotypic, molecular, and in-silico analyses. Specifically, we investigated the effects of MBF on cell viability, anchorage-dependent and-independent growth, migration, apoptosis, mitochondrial function, oxidative stress, cellular senescence, and proliferation, followed by gene expression, metabolomic, network pharmacology, and molecular docking analysis to discover the underlying mechanism of action. Our findings demonstrate that MBF exerts potent anti-proliferative and pro-apoptotic effects in BC cells, accompanied by metabolic alterations and modulation of cancer-associated signaling networks. Collectively, these results provide the first evidence supporting the repurposing potential of MBF as a candidate therapeutic agent for BC.

## 2. Results

### 2.1. MBF Reduces Cell Viability and Exhibits Potent Cytotoxicity

The impact of MBF on the viability of MCF-7 and MDA-MB-231 cells was determined using the MTT assay. Results showed that MBF treatment significantly reduced cell viability in both lines at 24 and 48 h in a dose-dependent manner (*p* < 0.05). For MCF-7 cells, the IC_50_ values were calculated as 377 μM and 93 μM at 24 and 48 h, respectively (Table 1). MDA-MB-231 cells exhibited even greater sensitivity, with values of 84 μM at 24 h and 18 μM at 48 h (Table 1). To evaluate the relative cytotoxic selectivity of MBF, its effect was also tested in non-malignant HEK-293 cells. In contrast, HEK-293 cells did not reach an IC_50_ within the tested range, with IC_50_ values remaining above 500 μM at both 24 and 48 h, suggesting lower sensitivity of this non-malignant control cell model to MBF under the present experimental conditions. MBF demonstrated greater in vitro cytotoxicity toward breast cancer cells than HEK-293 cells under the experimental conditions used in this study.

### 2.2. MBF Suppressed Anchorage-Dependent Clonogenic Potential

**Figure 1:**
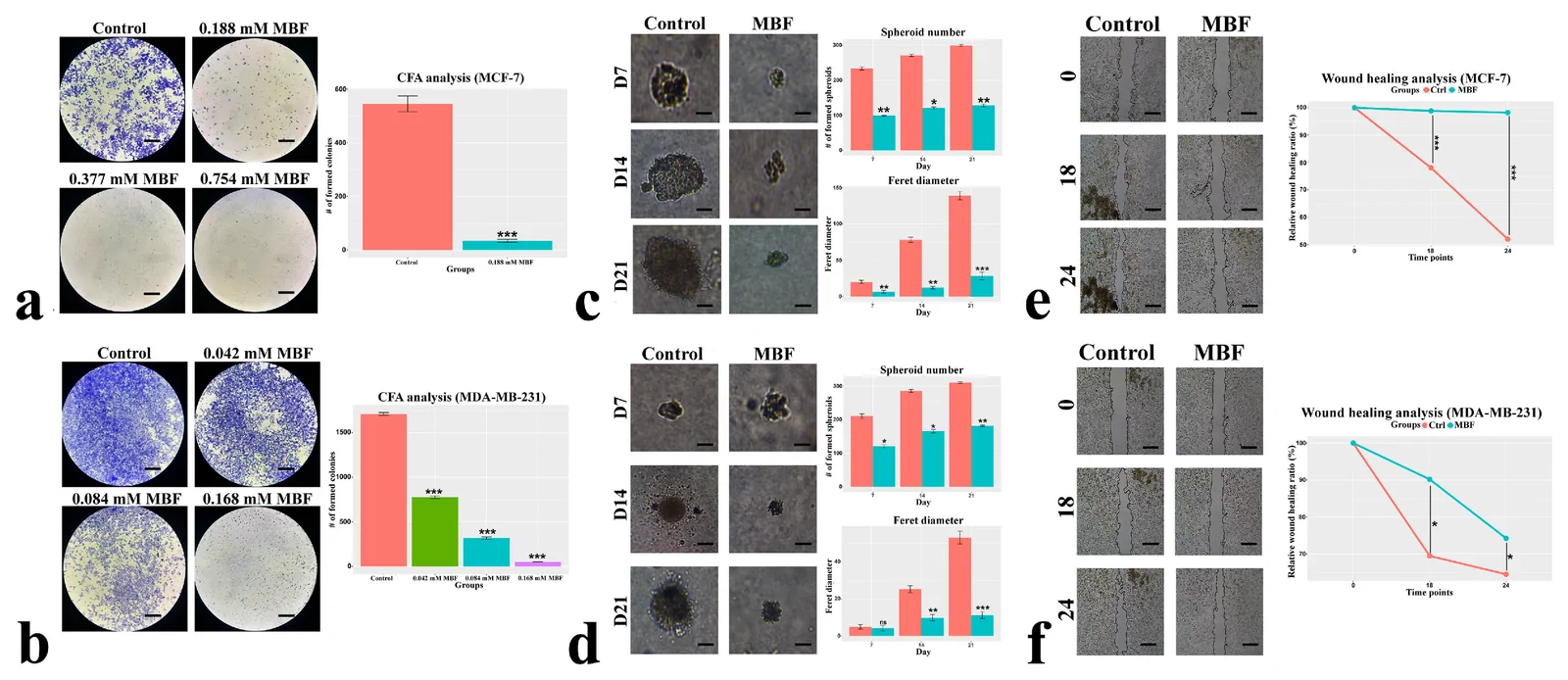
MBF’s impact on long-term anchorage-dependent and -independent growth and migration. Representative images and quantitative analysis of crystal violet-stained anchorage-dependent colony outgrowth in (**a**) MCF-7 and (**b**) MDA-MB-231 cells following MBF treatment. Representative formed spheroids and analysis of (**c**) MCF-7 and (**d**) MDA-MB-231 cells upon MBF administration. The orange bars represent control and the blue bars represent MBF-treated groups. Representative wound healing images and line plot analysis of (**e**) MCF-7 and (**f**) MDA-MB-231 cells. MBF; marbofloxacin. CFA; colony formation assay. D; day. ns; non-significant. * *p*-value < 0.05. ** *p*-value < 0.01. *** *p*-value < 0.001. Scale bars represent 50 µm in all representative images. The images were taken at 4× magnification. Data are presented as the mean ± SD of three independent biological replicates.

To evaluate the long-term effects of MBF on anchorage-dependent cellular outgrowth, a crystal violet-based colony outgrowth assay was performed. In MCF-7 cells, all MBF concentrations (1/2 IC_50_, IC_50_, and 2× IC_50_) led to significant crystal violet-stained growth area inhibition; specifically, colony outgrowth was completely abolished at IC_50_ and 2× IC_50_ doses, while the 1/2 IC_50_ dose resulted in 93.83% inhibition (*p* < 0.001) (Figure 1a). Similarly, MDA-MB-231 cells showed a reduction in the crystal violet-stained growth area by 54.61%, 81.39%, and 97.04% at the 1/2 IC_50_, IC_50_, and 2× IC_50_ doses, respectively (*p* < 0.001) (Figure 1b). These findings indicate that MBF markedly impaired long-term anchorage-dependent cellular outgrowth and culture expansion.

### 2.3. MBF Attenuated the Anchorage-Independent Growth and Spheroid Characteristics

The effect of MBF on tumorigenicity was further examined via soft agar assay. In MCF-7 cells, MBF treatment significantly reduced the average spheroid count by approximately 55–57% and the Feret diameter by 66.7–84.4% across 7-, 14-, and 21-day time points (*p* < 0.05) (Figure 1c). In MDA-MB-231 cells, spheroid counts decreased by roughly 42% at all time points, while the Feret diameter showed a significant reduction of up to 78.9% by day 21 (*p* < 0.05) (Figure 1d). These results demonstrate that MBF treatment effectively limits the three-dimensional growth and anchorage-independent survival potential essential for tumor progression.

### 2.4. MBF Exhibited a Strong Anti-Migrative Impact

The migratory capacity of BC cells was assessed using a wound-healing assay. In MCF-7 cells, the closed wound area was reduced 1.4-fold at 18 h and 2.2-fold at 24 h following MBF treatment (*p* < 0.001) (Figure 1e). For MDA-MB-231 cells, the closed wound area decreased 1.5-fold at 18 h and 1.2-fold at 24 h (*p* < 0.01) (Figure 1f). This indicates that MBF possesses a potent anti-migrative effect, potentially hindering the metastatic movement of breast cancer cells.

### 2.5. MBF Induced Apoptotic Cell Death in BC Cells

**Figure 2:**
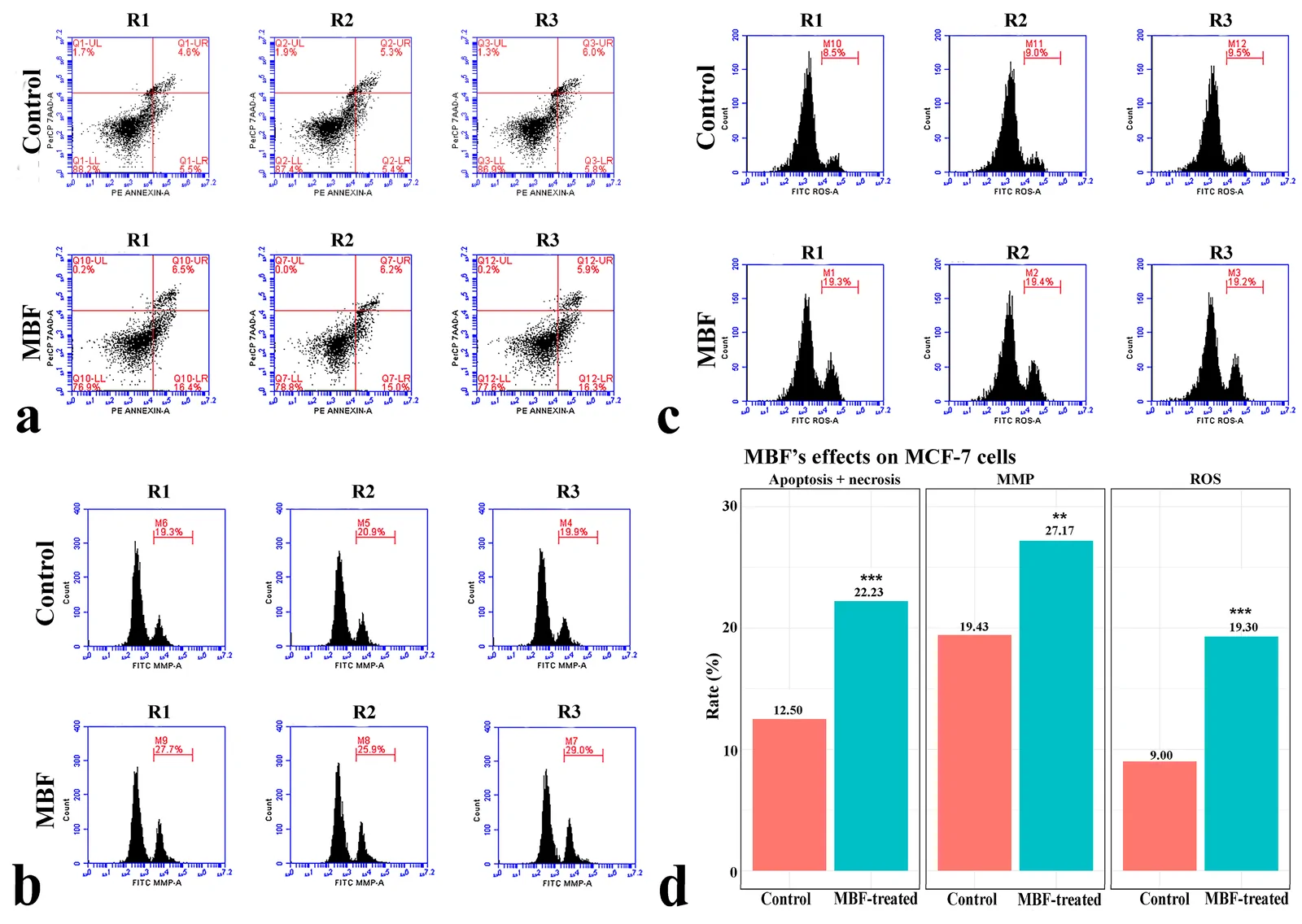
The examination of programmed cell death, mitochondrial membrane potential, and reactive oxygen species production in MCF-7 cells after MBF administration. (**a**) Flow cytometry plots of Annexin V/7AAD-stained MCF-7 cells. Cells in the upper right quadrant were classified as late apoptotic, those in the upper left quadrant as necrotic, those in the lower right quadrant as early apoptotic, and those in the lower left quadrant as viable cells. (**b**) Assessment of mitochondrial membrane potential (ΔΨm) by JC-1 staining. (**c**) Intracellular reactive oxygen species (ROS) levels measured by DCFH-DA staining. (**d**) The statistical analysis of flow cytometry plots. MBF; marbofloxacin, R; replica. ROS; reactive oxygen species. ** *p*-value < 0.01. *** *p*-value < 0.001. Data are presented as the mean ± SD of three independent biological replicates.

**Figure 3:**
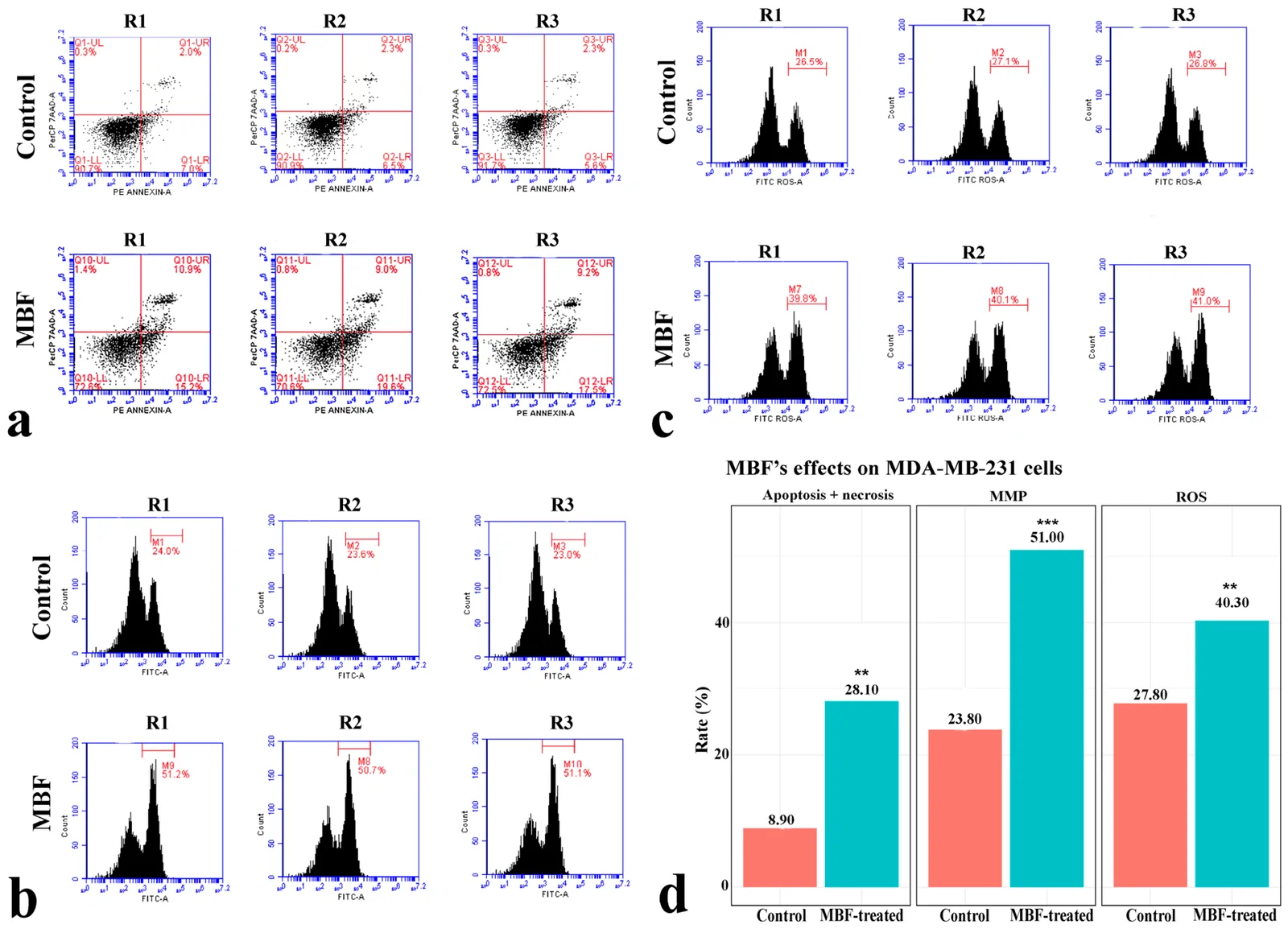
Effects of MBF on apoptosis, mitochondrial membrane potential, and reactive oxygen species generation in MDA-MB-231 cells. (**a**) The flow cytometry plots of control and MBF-treated cells. Cells in the upper right quadrant were classified as late apoptotic, those in the upper left quadrant as necrotic, those in the lower right quadrant as early apoptotic, and those in the lower left quadrant as viable cells. The histogram graphics of the cells on (**b**) ΔΨm and (**c**) ROS generation. (**d**) The bar charts of the analyzed data. MBF; marbofloxacin. R; replica. ** *p*-value < 0.01. *** *p*-value < 0.001. Data are presented as the mean ± SD of three independent biological replicates.

MBF’s effect on cellular death was evaluated by Annexin V/7AAD double staining protocol. MBF treatment significantly increased the number of apoptotic cells by 77.84% in MCF-7 cells (*p* < 0.001) (Figure 2a,d) and by 215.79% in MDA-MB-231 cells (*p* < 0.01) (Figure 3a,d). These data indicate that the reduction in cell population by MBF is driven by the induction of programmed cell death.

### 2.6. MBF Caused Mitochondrial Dysfunction and Elevated Oxidative Stress in BC Cells

The physiological state of the cells post-MBF treatment was further investigated by measuring mitochondrial membrane potential (ΔΨm) and reactive oxygen species (ROS) production. JC-1 staining demonstrated a significant increase in the proportion of cells exhibiting mitochondrial membrane depolarization following MBF treatment. The percentage of depolarized cells increased from 19.43% to 27.17% in MCF-7 cells and from 23.80% to 51.00% in MDA-MB-231 cells (*p* < 0.001). These findings indicate a loss of ΔΨm following MBF exposure. Concurrently, ROS production increased by 114.4% in MCF-7 (*p* < 0.01) (Figure 2c,d) and 44.9% in MDA-MB-231 cells (*p* < 0.001) (Figure 3c,d). This suggests that MBF-induced apoptosis is associated with mitochondrial pathway activation and the accumulation of intracellular oxidative stress.

### 2.7. MBF Reduced DNA Synthesis and Cellular Proliferation

**Figure 4:**
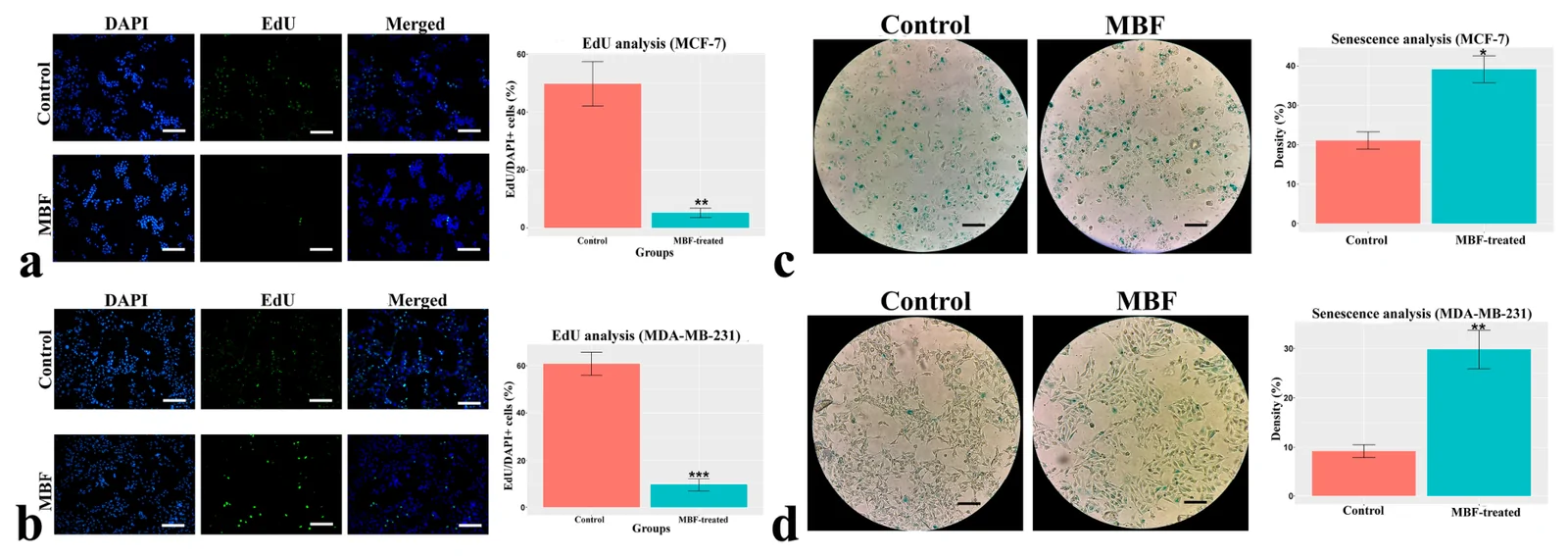
The cellular proliferation and senescence of BC cells after MBF treatment. The representative fluorescence microscopy images and bar chart analysis of (**a**) MCF-7 and (**b**) MDA-MB-231 cells. The fluorescent blue dots represent cell nuclei. The fluorescent green dots represent dividing cells at the S-phase. The representative figures and bar chart analysis of senescence-associated-ß-galactosidase-(SA-ß-gal)-stained (**c**) MCF-7 and (**d**) MDA-MB-231 cells. The blue-stained cells confirm the cellular senescence induced by MBF. MBF; marbofloxacin. DAPI; 4′,6-diamidino-2-phenylindole. EdU; 5-ethynyl-2′-deoxyuridine. Scale bar: 50 µm. * *p*-value < 0.05. ** *p*-value < 0.01. *** *p*-value < 0.001. The images were taken at 20× magnification. Scale bars represent 50 µm in all representative images. Data are presented as the mean ± SD of three independent biological replicates.

To determine the rate of cell proliferation upon MBF treatment, an EdU incorporation assay was conducted. Following 24 h of MBF treatment, the proportion of EdU-positive cells decreased by 89.58% in MCF-7 cells (*p* < 0.01) (Figure 4a) and by 84.39% in MDA-MB-231 cells (*p* < 0.001) (Figure 4b). The marked decrease in EdU incorporation confirms that MBF effectively halts active DNA synthesis and cellular proliferation.

### 2.8. MBF Promoted Cellular Senescence

The effect of MBF on cellular senescence was evaluated using SA-ß-gal staining assay. MBF treatment induced a significant increase in senescent cells, with an 85.7% increase in MCF-7 (*p* < 0.05) (Figure 4c) and a 223.9% increase in MDA-MB-231 cells (*p* < 0.01) (Figure 4d). These findings suggest that MBF contributes to long-term growth arrest by driving BC cells into a state of irreversible cellular senescence.

### 2.9. Differential Gene Expression Profiles Reflect MBF-Induced Growth Arrest and Apoptosis While Inhibiting Proliferation and Cell Cycle Progression

Gene expression analysis revealed significant molecular alterations following MBF treatment. In MCF-7 cells, expression of *ANKRD1* (10.29-fold, *p* < 0.001), *BIRC5* (2-fold, *p* < 0.001), *CCND1* (2.66-fold, *p* < 0.001), *CCNE1* (3.30-fold, *p* < 0.001), *CDK6* (2.8-fold, *p* < 0.001), *MKI67* (1.76-fold, *p* < 0.01), and *MMP9* (4.54-fold, *p* < 0.001) was significantly downregulated, while *CDKN1A* (4-fold, *p* < 0.001) and *PUMA* (2-fold, *p* < 0.01) was upregulated. In MDA-MB-231 cells, *ANKRD1* (9.35-fold, *p* < 0.001), *BCL2* (2.23-fold, *p* < 0.01), *CSF2* (1.87-fold, *p* < 0.05), *EDN1* (3.89-fold, *p* < 0.01), and *MKI67* (2.96-fold, *p* < 0.05) were downregulated, whereas *CDKN1A* (3.71-fold, *p* < 0.01) was significantly upregulated. This molecular shift indicates that MBF activates pathways favoring cell cycle arrest and apoptosis while suppressing genes essential for cell survival and proliferation (Table 2).

### 2.10. MBF Modulated Metabolic Landscape and Altered Signaling Pathways in BC Cells

To further elucidate the biochemical underpinnings of the anti-proliferative effects of MBF, untargeted metabolomic profiling was conducted on both MCF-7 and MDA-MB-231 BC cell lines. Our findings demonstrate that MBF treatment triggers a profound metabolic shift, characterized by the suppression of survival-linked pathways and the induction of phase-II detoxification mechanisms.

**Figure 5:**
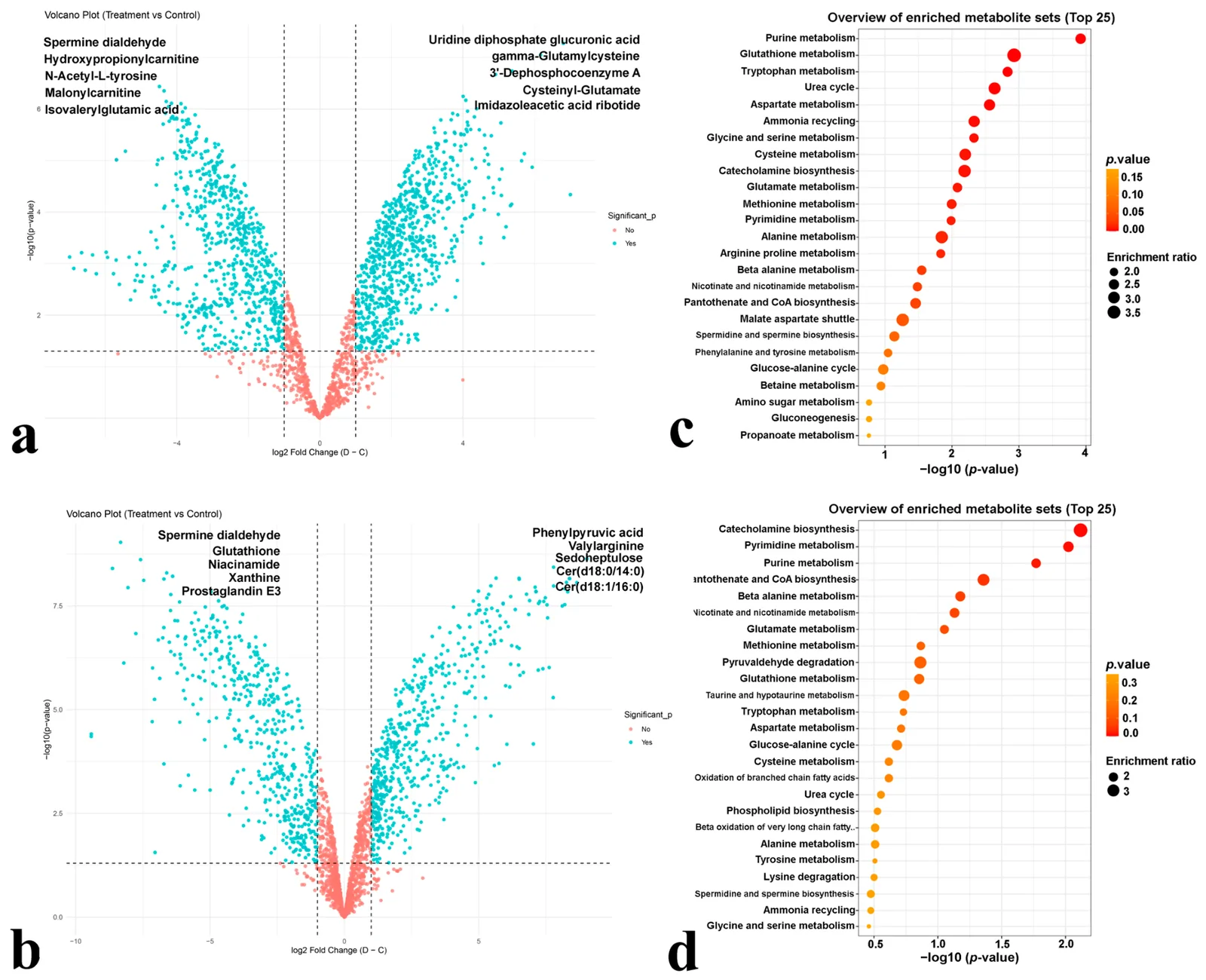
Untargeted metabolomic profiling of MBF-treated cells. (**a**) Volcano plot showing significantly altered metabolites in MBF-treated MCF-7 and (**b**) MDA-MB-231 cells compared to untreated controls. Metabolic pathway enrichment analysis of differentially abundant metabolites identified in (**c**) MCF-7 and (**d**) MDA-MB-231 cells.

In MCF-7 cells, MBF treatment significantly altered the abundance of metabolites involved in phase II conjugation, glutathione synthesis, and specialized receptor signaling. The top five upregulated (FC > 1.5, *p* < 0.05) metabolites were found to be uridine diphosphate glucuronic acid (UDP–glucuronic acid), *γ*-glutamylcysteine, 3′-dephosphocoenzyme A, cysteinyl–glutamate, and imidazoleacetic acid ribotide. Conversely, treated MCF-7 cells exhibited a marked depletion of organic acids, amino acid derivatives, and polyamine oxidation products, with the top five downregulated metabolites identified as isovalerylglutamic acid, malonylcarnitine, *N*-acetyl-L-tyrosine, hydroxypropionylcarnitine, and spermine dialdehyde (Figure 5a). These shifts were associated with changes in purine metabolism, glutathione metabolism, tryptophan metabolism, the urea cycle, aspartate metabolism, and the UDP–glucuronate/phase II detoxification pathways, indicating extensive metabolic modulation involving nucleotide turnover, redox homeostasis, amino acid metabolism, and energy-related pathways (Figure 5c).

In MDA-MB-231 cells, characterized by their aggressive phenotype, MBF treatment induced profound metabolic remodeling through distinct lipid-associated metabolic shift. The top five upregulated endogenous metabolites were dominated by pro-apoptotic ceramides, pentose intermediates, and amino acid products, identified as ceramide d18:1/16:0 [Cerd18:1/16:0)], ceramide d18:0/14:0 [Cer(d18:0/14:0)], sedoheptulose, valylarginine, and phenylpyruvic acid. Additionally, MBF-treated MDA-MB-231 cells displayed a significant depletion of anti-inflammatory lipids, purines, and antioxidants. The top five downregulated metabolites in this line were prostaglandin E3, xanthine, niacinamide, glutathione (reduced), and spermine dialdehyde (Figure 5b).

Pathway enrichment analysis indicated that these alterations primarily impact pyrimidine metabolism, purine metabolism, pantothenate and coenzyme A biosynthesis, and energy-associated pathways (Figure 5d). Collectively, these findings demonstrate that MBF profoundly remodels the metabolic landscape of MDA-MB-231 cells by perturbing lipid metabolism, redox homeostasis, nucleotide metabolism, and mitochondrial energy-related pathways.

Comparative metabolomic analysis of the two cell lines revealed several convergent metabolic vulnerabilities exploited by MBF treatment. In both lineages, a significant depletion of polyamine oxidation products, specifically spermine dialdehyde, was observed, indicating a direct impairment of tumor cell proliferation and macromolecular translation. Furthermore, MBF treatment resulted in marked nucleotide biosynthesis dysfunction, characterized by the enrichment and disruption of purine metabolism in MCF-7 cells, and both pyrimidine and purine pathways in MDA-MB-231 cells.

Despite these shared vulnerabilities, distinct, lineage-specific metabolic shifts were identified between the two models. In the ER+ MCF-7 cells, MBF treatment triggered a prominent phase II conjugation response, characterized by the significant accumulation of UDP–glucuronic acid, which is highly associated with extrahepatic steroid metabolism in ER+ breast cancer models. Rather than suffering direct antioxidant depletion, MCF-7 cells exhibited a compensatory upregulation of glutathione synthesis intermediates, specifically *γ*-glutamylcysteine and cysteinyl–glutamate, likely as an adaptive response to counteract MBF-induced oxidative stress. This was accompanied by a significant depletion of organic acids and acylcarnitines, including malonylcarnitine and hydroxypropionylcarnitine.

Conversely, the metabolic reprogramming in the triple-negative MDA-MB-231 model was characterized by a profound lipid-centric shift and antioxidant system collapse. MBF treatment induced a significant accumulation of pro-apoptotic sphingolipids, along with the accumulation of sedoheptulose, pointing to a redirection of glucose flux through the non-oxidative branch of the pentose phosphate pathway. Crucially, MDA-MB-231 cells failed to sustain their redox buffer system, suffering a critical depletion of reduced glutathione and niacinamide, which directly supports the observed mitochondrial outer membrane depolarization and elevated apoptotic rate. These results indicate that the anti-cancer activity of MBF is driven by a multi-targeted metabolic shift that effectively impairs cellular antioxidant capacity, redox homeostasis, and macromolecular synthesis across different breast cancer subtypes.

### 2.11. Network Pharmacology Revealed MBF’s Potential Molecular Targets and Signaling Pathways

**Figure 6:**
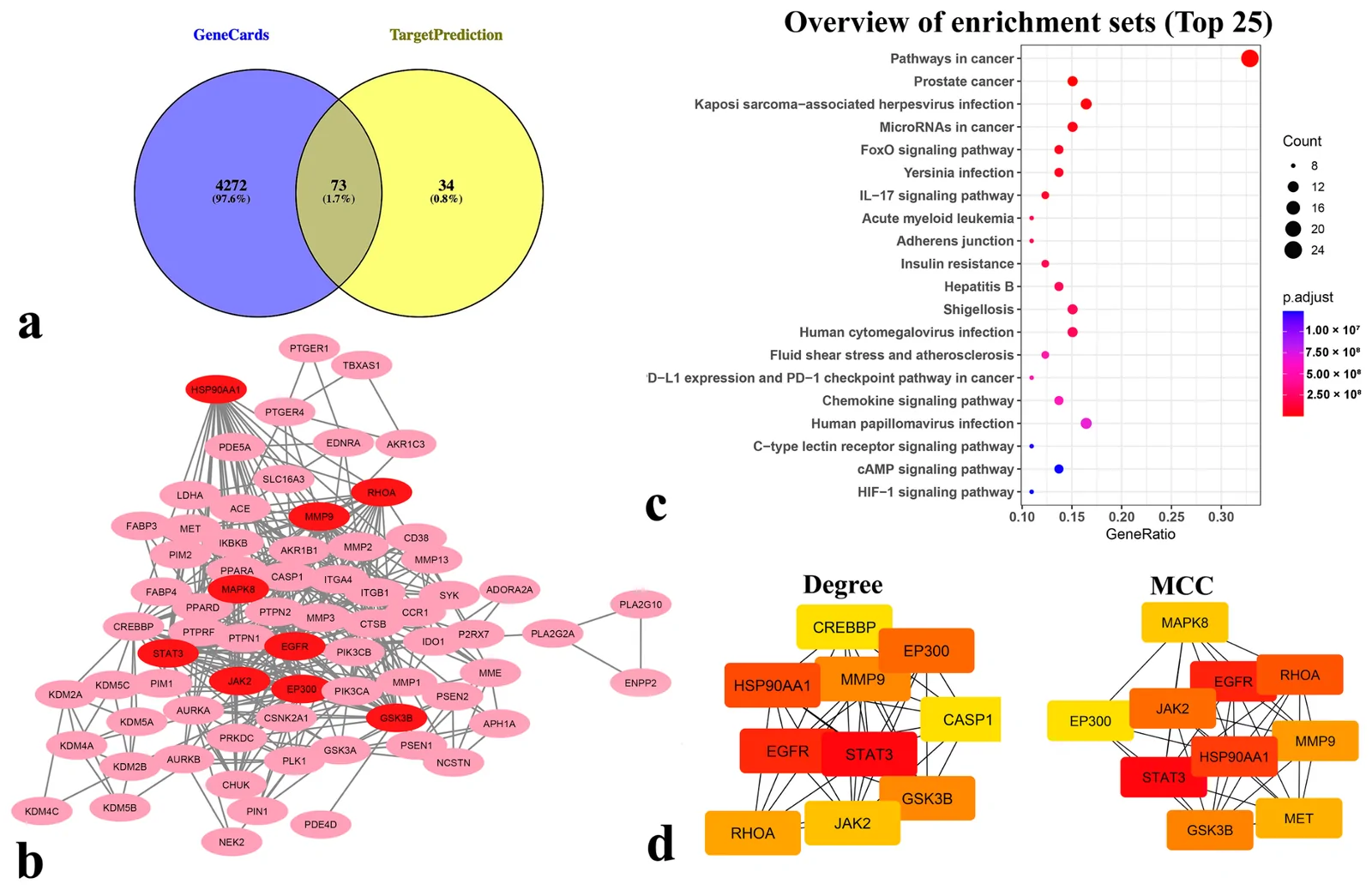
Network pharmacology analysis of MBF in BC. (**a**) Venn diagram representing the overlapped genes between putative MBF targets and BC-associated ones. (**b**) Protein–protein interaction (PPI) network of the 73 overlapping targets. Common hub genes identified by both Degree and Maximal Clique Centrality (MCC) analyses are highlighted in red. (**c**) KEGG pathway enrichment analysis of the common targets. (**d**) Top-10-ranked hub genes identified using the Degree and MCC algorithm.

To further exploit molecular targets of MBF in BC, a network pharmacology approach was employed. SwissTargetPrediction identified 107 putative targets of MBF, while BC-related ones were retrieved from the GeneCards database. Venn analysis identified 73 overlapping genes, which were considered potential therapeutic targets of MBF in BC (Figure 6a).

The 73 common targets were subsequently analyzed using the STRING database to generate a protein-protein interaction (PPI) network. Network visualization in Cytoscape demonstrated extensive interactions among the identified targets (Figure 6b). Several high-confidence interactions were identified within the network, including EGFR–STAT3, JAK2–STAT3, EP300–STAT3, EGFR–HSP90AA1, and CREBBP–EP300, highlighting the presence of interconnected signaling modules involved in cancer progression and cellular survival.

KEGG pathway enrichment analysis revealed significant enrichment of cancer-related signaling pathways, including the following: pathways in cancer, FoxO signaling pathway, IL-17 signaling pathway, and PD-L1 expression and PD-1 checkpoint pathway in cancer (Figure 6c). These findings suggest that MBF may exert its anticancer effects through the coordinated modulation of multiple oncogenic pathways involved in cell proliferation, survival, migration, and metastasis.

To identify key regulatory nodes within the PPI network, topological analyses were performed using both the Degree and MCC algorithms in the CytoHubba plugin. Several genes, including EGFR, STAT3, HSP90AA1, EP300, JAK2, MMP9, MAPK8, RHOA, and GSK3B, were consistently identified as hub genes by both methods (Figure 6b), suggesting their potential central roles in the predicted anticancer mechanisms of MBF. STAT3 emerged as the highest-ranked hub gene, exhibiting a Degree score of 38 and an MCC score of 228,872, followed by EGFR (Degree = 35; MCC = 228,547), HSP90AA1 (Degree = 29; MCC = 191,940), EP300 (Degree = 28; MCC = 82,512), GSK3B (Degree = 26; MCC = 103,223), and MMP9 (Degree = 26; MCC = 98,216) (Figure 6d). Additional highly ranked nodes included RHOA (Degree = 22; MCC = 125,354) and JAK2 (Degree = 21; MCC = 119,082). Genes consistently ranked among the top candidates by both algorithms were considered robust hub genes and prioritized for subsequent molecular docking analyses.

### 2.12. MBF Exhibits Favorable Binding Affinity Toward HSP90AA1 and EGFR

**Figure 7:**
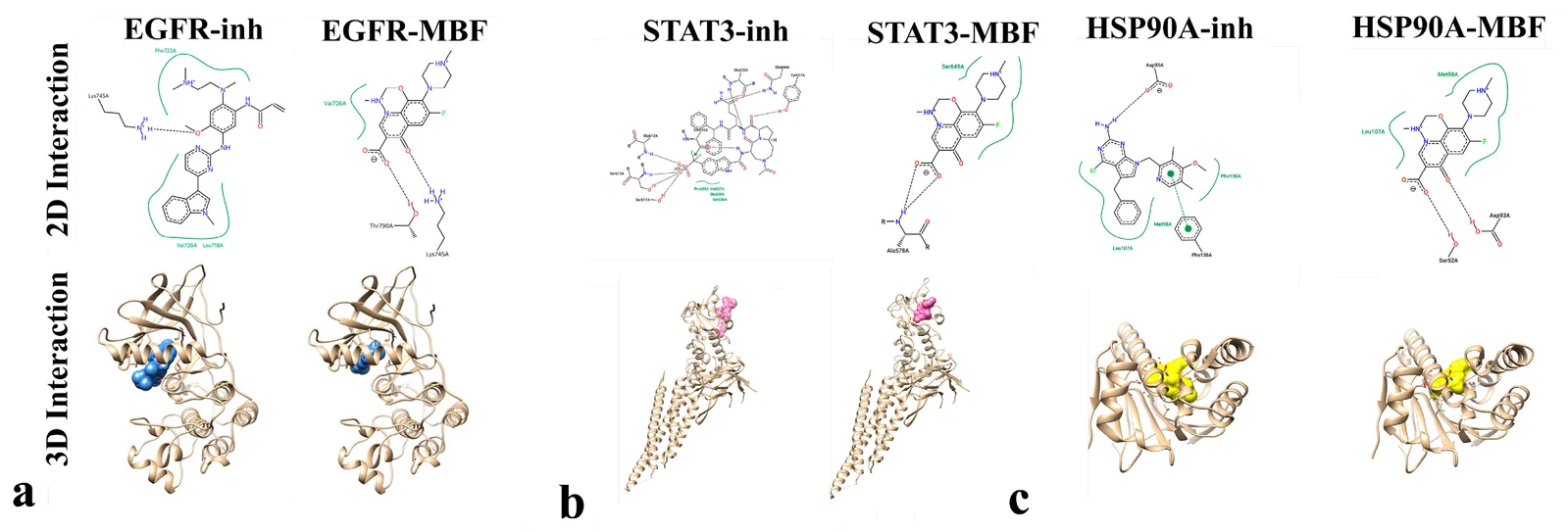
Molecular docking analysis of MBF against selected hub proteins identified through network pharmacology. (**a**) Two-dimensional (2D) and three-dimensional (3D) binding interactions of MBF and the corresponding reference inhibitor, osimertinib, within the EGFR binding pocket. (**b**) 2D and 3D binding interactions of MBF and the corresponding reference inhibitor, SI109, within the STAT3 binding site. (**c**) 2D and 3D binding interactions of MBF and the corresponding reference inhibitor, 4-chloranyl-7-[(4-methoxy-3,5-dimethyl-pyridin-2-Yl)methyl]-5-(phenylmethyl)pyrrolo[2,3-D]pyrimidin-2-amine, within the HSP90A ATP-binding pocket. Predicted binding poses and key amino acid interactions are illustrated to compare the binding behavior of MBF with established target-specific inhibitors. EGFR; epidermal growth factor receptor. STAT3; signal transducer and activator of transcription 3. HSP90A; heat shock protein 90 alpha family. MBF; marbofloxacin. inh; inhibitor of each corresponding protein.

Molecular docking was performed to evaluate the binding affinity and interaction profiles of MBF with cancer-related target proteins, namely EGFR, STAT3, and HSP90A (Figure 7).

The docking results were compared with those of the respective co-crystallized reference inhibitors (Table 3).

For EGFR, the reference inhibitor osimertinib demonstrated a binding affinity of −7.6 kcal/mol, with hydrogen bonds involving Lys745A and hydrophobic interactions with Phe723A, Val726A, and Leu718A. MBF exhibited a comparable binding affinity of −7.2 kcal/mol, forming a hydrogen bond with Thr790A and Lys745A, and hydrophobic interactions with Val726A. The RMSD values for marbofloxacin ranged from 2.822 to 4.018 Å.

For STAT3, the reference inhibitor SI109 showed a binding affinity of −7.3 kcal/mol and formed seven hydrogen bonds with Tyr657A, Gln644A, Glu638A, Ser636A, Glu612A, Ser613A, and Ser611A, together with hydrophobic contacts involving Pro639A, Val637A, Glu638A, and Ser636A. MBF displayed a weaker binding affinity of −5.4 kcal/mol, forming a single hydrogen bond with Ala578A and one hydrophobic interaction with Ser649A. RMSD values ranged from 3.481 to 5.715 Å, indicating moderate deviation from the reference binding conformation.

Among all investigated targets, HSP90A exhibited the strongest interaction with MBF. The reference inhibitor showed a binding affinity of −10.1 kcal/mol, forming hydrogen bonds with Asp93A and Phe138A and hydrophobic interactions with Leu107A, Met98A, and Phe138A. MBF demonstrated a relatively high binding affinity of −8.4 kcal/mol, with hydrogen bonds involving Asp93A and Ser52A and hydrophobic contacts with Met98A and Leu107A. Notably, both the inhibitor and MBF exhibited RMSD values of 0 Å, indicating highly stable docking conformations.

Overall, MBF demonstrated the strongest binding toward HSP90A (−8.4 kcal/mol), followed by EGFR (−7.2 kcal/mol), whereas weaker interactions were observed with STAT3 (−5.4 kcal/mol). The docking analysis suggests that HSP90A and EGFR may represent the most favorable molecular targets for MBF among the proteins evaluated.

### 2.13. Root Mean Square Fluctuation (RMSF)-Based Protein Flexibility Analysis Supports the Stability of MBF–EGFR and MBF–HSP90A Complexes

**Figure 8:**
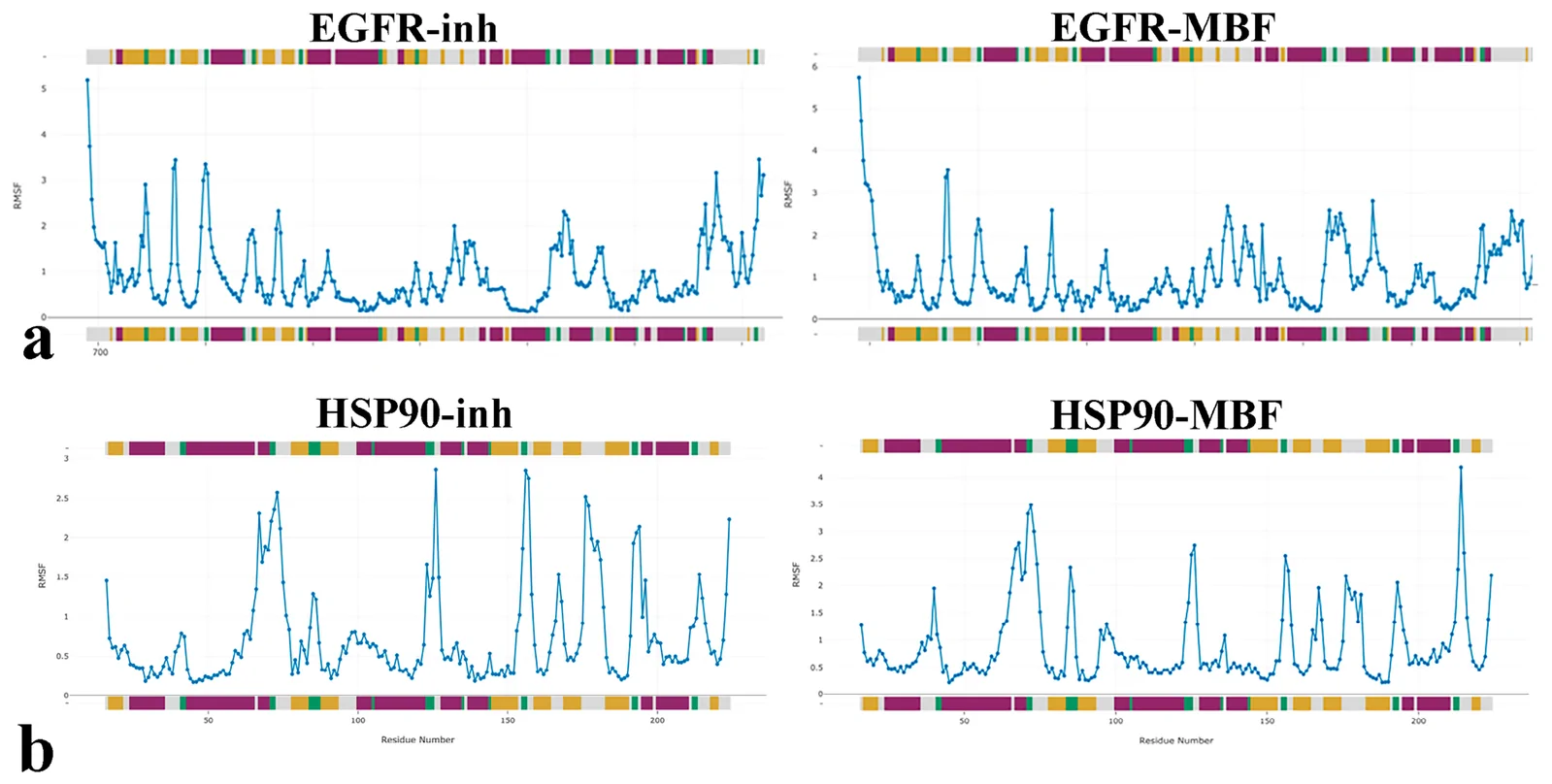
RMSF analysis of reference inhibitor- and MBF-bound protein complexes during molecular dynamics simulations. (**a**) Residue flexibility profiles of EGFR in complex with the reference inhibitor osimertinib (EGFR-inh) and MBF (EGFR-MBF). (**b**) Residue flexibility profiles of HSP90A in complex with the reference inhibitor 4-chloranyl-7-[(4-methoxy-3,5-dimethyl-pyridin-2-Yl)methyl]-5-(phenylmethyl)pyrrolo[2,3-D]pyrimidin-2-amine (HSP90-inh) and MBF (HSP90-MBF). EGFR; epidermal growth factor receptor. HSP90A; heat shock protein 90 alpha family. MBF; marbofloxacin. inh; inhibitor.

The residue-level flexibility of EGFR and HSP90A complexes was evaluated through RMSF analysis during the molecular dynamics simulation (Figure 8). RMSF values provide insight into the mobility of amino acid residues and the structural stability of protein–ligand complexes. In EGFR complexes, marbofloxacin binding preserves the global structural dynamics of EGFR similarly to the reference inhibitor, osimertinib. Higher flexibility is observed primarily at the N-terminal region (approximately residues 690–700) and at several loop regions around residues 730–750, 850–880, 910–930, and 980–1010, whereas residues forming α-helices and β-sheets generally display lower RMSF values (<1 Å), indicating greater structural rigidity. The MBF-bound complex shows slightly higher fluctuations at several loop regions, particularly around residues 860–880 and 915–930, while the overall magnitude and distribution of residue mobility remain comparable to those of the osimertinib-bound complex. These findings suggest that MBF does not induce substantial conformational destabilization of the EGFR kinase domain during the simulation.

In the HSP90A complexes, lower overall RMSF values were observed compared with EGFR, indicating greater structural rigidity. The HSP90A–inhibitor complex displayed RMSF values predominantly below 1.0 Å, with moderate fluctuations occurring approximately at residues 65–75, 120–130, 155–160, and 175–185, where peaks reached 2.5–3.0 Å. Similarly, the HSP90A–MBF complex maintained low fluctuations throughout most of the simulation trajectory. However, several regions exhibited increased mobility, particularly around residues 65–75 and 210–220, with maximum RMSF values reaching approximately 3.5–4.2 Å. Notwithstanding these localized fluctuations, the overall RMSF profile remained relatively low, indicating stable ligand binding and preservation of protein structural integrity.

Comparison of these inspected systems revealed that the HSP90A complexes exhibited lower residue fluctuations than the EGFR complexes, suggesting greater conformational stability during simulation. Furthermore, the RMSF profile of the HSP90A–MBF complex closely resembled that of the reference inhibitor, supporting the docking results that identified HSP90A as the most favorable target for MBF.

## 3. Discussion

FQs were originally developed as broad-spectrum antibiotics used to treat severe bacterial infections, including urinary tract infection and sexually transmitted diseases, by exploiting its inhibitory impact on bacterial DNA transcription either by targeting DNA gyrase or topoisomerase-II [17]. Considering the high financial burden and time-consuming processes involved in the development of new anticancer agents, FQs have recently emerged as promising candidates for drug repurposing owing to their potential anticancer activities across various malignancies [18]. Given the growing interest in drug repurposing, this study was designed to characterize the anti-neoplastic efficacy of MBF on BC cells for the first time, to the best of our knowledge.

Our findings indicate that MBF induced a potent, dose-dependent inhibition of BC cell growth and displayed greater cytotoxicity toward BC cells than HEK-293 cells. Although these cells do not represent normal mammary epithelium, they were included as a commonly used non-malignant human cell model. Specifically, MDA-MB-231 cells demonstrated superior sensitivity to MBF relative to other FQs previously documented in the literature [19]. This anti-proliferative efficacy is corroborated by a significant reduction in EdU incorporation, indicating a sustained impairment of long-term proliferative capacity rather than an exclusive reliance on cytotoxic mechanisms. Furthermore, the substantial increase in the proportion of SA-β-gal-positive cells suggests that MBF-induced growth arrest is perpetuated through the induction of irreversible cellular senescence. Complementary colony formation and spheroid assays further substantiated the attenuation of long-term proliferative capacity. These data align well with the previously reported capacity of other FQs, including ciprofloxacin (CIP), levofloxacin (LEV), and nano-encapsulated CIP, to suppress the long-term tumorigenicity of cancer cells [20,21].

Although several of the effective in vitro concentrations observed in the present study exceed the plasma concentrations typically achieved following conventional veterinary administration of MBF, the translational implications appear to differ between breast cancer subtypes. Pharmacokinetic studies have reported peak plasma concentrations (Cmax) ranging from approximately 1.4 to 6.3 μg/mL (≈3.9–17.3 μM) depending on the animal species, dose, and route of administration [12,22,23,24]. Notably, the lowest IC_50_ identified in the present study (18 μM in MDA-MB-231 cells after 48 h) is comparable to the upper range of reported plasma exposure, whereas substantially higher concentrations were required to inhibit MCF-7 cells. Therefore, our findings should primarily be interpreted as proof-of-concept demonstrating the anticancer activity of MBF in vitro. Future studies investigating optimized formulations, targeted drug delivery systems, combination therapies, or local drug accumulation within tumors will be required to determine whether therapeutically relevant concentrations can be achieved in vivo.

Interestingly, a greater sensitivity to MBF in MDA-MB-231 cells was noted along both treatment durations. This differential response may, at least in part, be associated with the distinct molecular and metabolic characteristics of the two subtypes. TNBC cells are characterized by elevated basal oxidative stress, enhanced metabolic plasticity, and a higher dependence on antioxidant defense systems to maintain redox homeostasis compared with hormone receptor-positive counterparts [25,26,27]. Consistently, several studies have reported that FQs exert stronger antiproliferative and pro-apoptotic impact on TNBC cells [19,28]. The extensive perturbations of redox homeostasis, lipid metabolism, nucleotide metabolism, and the accumulation of pro-apoptotic ceramides together with the marked depletion of glutathione observed in MDA-MB-231 cells further supports the possibility of heightened sensitivity to MBF treatment.

The observed increase in ROS production together with the loss of ΔΨm and the stimulation of apoptotic cell death following MBF treatment may indicate an activation of oxidative stress-associated apoptotic responses in BC cells. These findings are consistent with previous reports demonstrating that CIP and its metal-complexed or Mannich base-modified derivatives promote apoptosis through mitochondrial dysfunction [29,30,31]. Similar pro-apoptotic effects have been shown to suppress BC growth by stimulating apoptosis and impairing mitochondrial biogenesis [32], while increased ROS production and apoptosis have been documented in lung adenocarcinoma [33]. Likewise, moxifloxacin (MFLX) and its derivatives were shown to promote apoptosis in several malignancies, such as prostate [34], colon [35], breast [36], melanoma [37], and pancreatic [38] cancers. Increased apoptotic cell populations have also been demonstrated following enoxacin (ENX) or lomefloxacin use in cervical [39], prostate [40], colon [41], melanoma [42], and leukemia [43] models.

Notably, the molecular alterations observed in the present study further support the apoptosis-inducing potential of MBF. The transcriptional activation of *PUMA* and suppression of *BIRC5* in MCF-7 cells, as well as the downregulation of *BCL-2* in MDA-MB-231 cells, are consistent with the induction of pro-apoptotic signaling pathways. Collectively, these findings suggest that MBF may exert its anticancer effects, at least in part, through oxidative stress-mediated mitochondrial dysfunction, ultimately promoting apoptosis in BC cells in a manner comparable to that reported for other members of the FQs.

To gain further mechanistic insight into the observed anticancer activity of MBF, we performed untargeted metabolomic profiling. The metabolomic analysis revealed substantial alterations in nucleotide metabolism, glutathione homeostasis, amino acid metabolism, polyamine metabolism, and coenzyme A biosynthesis. Rather than reflecting a generalized metabolic collapse, the metabolomic alterations suggest that MBF elicits subtype-specific metabolic adaptations while consistently perturbing pathways essential for cellular proliferation and redox regulation. Specifically, the depletion of spermine dialdehyde and the subsequent dysfunction in purine and pyrimidine metabolism, which cancer cells rely on to sustain reproductive integrity and rapid replication [44,45], suggest that MBF suppresses anabolic growth of BC cells. This phenomenon directly underpins the observed 84.39% to 89.58% reduction in EdU incorporation, the transition into cellular senescence, and the significant downregulation of *MKI67*, an important proliferation marker, in both cells.

Interestingly, the metabolomic responses differed substantially between the two breast cancer subtypes. In MCF-7 cells, MBF treatment resulted in significant accumulation of UDP–glucuronic acid, *γ*-glutamylcysteine, cysteinyl–glutamate, and 3′-dephosphocoenzyme A, accompanied by enrichment of glutathione metabolism, purine metabolism, glycine and serine metabolism, cysteine metabolism, and pantothenate and coenzyme A biosynthesis. UDP–glucuronic acid serves as the obligate co-substrate for UDP–glucuronosyltransferases involved in phase II detoxification [46], whereas *γ*-glutamylcysteine and cysteinyl–glutamate are closely associated with glutathione biosynthesis and antioxidant defense [47] and reflect activation of a compensatory antioxidant response to the severe oxidative and mitochondrial stress induced by the MBF treatment [48]. The accumulation of these metabolites therefore suggests activation of compensatory detoxification and antioxidant pathways in response to MBF-induced metabolic stress. Likewise, increased levels of 3′-dephosphocoenzyme A indicate perturbation of coenzyme A metabolism [49,50], which may reflect altered mitochondrial energy homeostasis. Collectively, these findings suggest that MCF-7 cells retain the capacity to mount adaptive metabolic responses that partially compensate for MBF-induced oxidative and metabolic stress, consistent with their relatively lower sensitivity to MBF.

In the more aggressive MDA-MB-231 model, this metabolic redirection is notably intensified, encompassing broader disruptions in pyrimidine metabolism, purine metabolism, pantothenate and coenzyme A biosynthesis, glutathione metabolism, and nicotinate and nicotinamide metabolism, accompanied by the accumulation of Cer(d18:1/16:0) and Cer(d18:0/14:0) together with significant depletion of glutathione, niacinamide, xanthine, and prostaglandin E3. Ceramides are well-established bioactive sphingolipids that regulate stress signaling, apoptosis, and mitochondrial dysfunction, whereas glutathione and niacinamide are central components of intracellular antioxidant and redox homeostasis [51,52,53]. Therefore, the concomitant accumulation of ceramides and depletion of antioxidant metabolites is consistent with severe metabolic stress and impaired redox buffering capacity in MBF-treated MDA-MB-231 cells. Although the present study does not establish a direct causal relationship between these metabolic alterations and apoptosis, the metabolomic profile is concordant with the observed increases in ROS production, mitochondrial membrane depolarization, apoptotic cell death, and cellular senescence.

These subtype-specific metabolic responses may also explain the greater susceptibility of MDA-MB-231 cells to MBF. Triple-negative breast cancer cells exhibit higher basal oxidative stress and increased dependence on antioxidant systems than hormone receptor-positive breast cancer cells [25,26,27]. Accordingly, while MCF-7 cells predominantly accumulated metabolites associated with detoxification and glutathione precursor synthesis, MDA-MB-231 cells exhibited depletion of glutathione together with widespread disruption of nucleotide metabolism, lipid metabolism, and cellular redox pathways, suggesting a reduced capacity to compensate for MBF-induced metabolic stress. Glutathione (GSH) represents one of the major intracellular antioxidant defenses responsible for maintaining redox balance and protecting cells from oxidative damage [54]. These findings are consistent with established evidence regarding the pro-oxidant activity of other FQs in various cancer models. It has been demonstrated that lomefloxacin decreases the level of cellular GSH in melanoma cells [42], while CIP similarly altered redox signaling in breast cancer cells by GSH depletion [19]. In a glioblastoma multiforme model, CIP and MFLX treatment resulted in GSH depletion coupled with the activation of caspase-3/7 and the induction of S- and sub-G1 cell cycle arrest [55]. These findings suggest that MBF may share metabolic features with other FQs, particularly regarding perturbation of antioxidant metabolism and redox homeostasis. Altogether, these metabolic alterations confirm that MBF does not merely act as a cytotoxic agent but causes metabolic alterations to favor growth arrest and apoptosis.

As a next step, we integrated a network pharmacology approach by overlapping BC- associated genes retrieved from GeneCards with putative MBF targets predicted through SwissTargetPrediction. With this method, a PPI network was constructed to identify the core targets involved in MBF’s anticancer activity on BC cells. Topological analysis of the network revealed several high-connectivity hub genes, including EGFR, STAT3, HSP90AA1, EP300, GSK3B, JAK2, MAPK8, MMP9, and RHOA, which are well-established regulators of tumor cell proliferation, survival, migration, invasion, and therapeutic resistance, suggesting that MBF may interfere with several oncogenic processes simultaneously [56,57,58,59,60,61,62,63,64,65,66,67,68]. Interestingly, several of the identified hub genes may also provide a mechanistic explanation for the anti-migratory effects of MBF. MMP9, a key mediator of extracellular matrix remodeling and metastasis [59], emerged as one of the central nodes within the PPI network. Our findings that *MMP9* expression was significantly downregulated in MBF-treated MCF-7 cells, while a significant inhibition of the wound closure capacity was noted in both cells, align with broader documentation evidencing that FQs such as CIP and LEV have been reported to suppress TGF-ß and PMA-induced MMP-9 production in lung adenocarcinoma cells [69]. Furthermore, the identification of RHOA, EGFR, and JAK2 as hubs is consistent with the possibility that MBF may interfere with the signaling cascades involved in cytoskeletal organization, cell motility, and invasion.

Among the identified hub targets, HSP90AA1 and EGFR emerged as particularly compelling candidates due to their central positions within the PPI network and their well-established roles in breast cancer progression [56,70,71]. HSP90AA1 is an inducible molecular chaperone that stabilizes a vast array of proteins, including EGFR and STAT3, preventing their proteasomal degradation [70,71]. High HSP90AA1 expression was found to be correlated with advanced tumor stage, poor prognosis, and distant metastasis in BC patients [72]. Recent studies revealed a novel role for HSP90AA1 in metabolic reprogramming through VDAC1 interaction [57]. This HSP90AA1-VDAC1 interplay contributes to BC progression and doxorubicin resistance by activating PI3K/AKT signaling [57]. In particular, HSP90AA1 knockdown in BC has been shown to significantly inhibit its proliferation, migration, and invasion, while inducing cell-cycle arrest [73]. Aberrant EGFR activation has been linked with cellular proliferation, migration, survival, metabolic adaptation, and therapeutic resistance through downstream signaling cascades such as PI3K/AKT, MAPK, and JAK/STAT pathways [74]. In particular, detected phenotypic alterations such as reduced proliferation, impaired migration, increased apoptosis, and profound metabolic alterations following MBF administration are consistent with the biological processes regulated by HSP90AA1 and EGFR signaling. Collectively, these observations are consistent with perturbation of HSP90AA1–EGFR-associated oncogenic networks.

To bridge the gap between network prediction and physical target interaction, molecular docking analyses were performed using selected hub proteins. Interestingly, MBF exhibited the strongest predicted binding affinity toward HSP90AA1, surpassing all other targets. Moreover, MBF retained several key interactions observed in the reference inhibitor complex, suggesting favorable accommodation within the HSP90 ATP-binding pocket. EGFR represented the second most favorable target, displaying a binding affinity comparable to that of the reference inhibitor and relatively low RMSF values, indicative of a stable binding orientation. In contrast, weaker binding affinities and less favorable interaction profiles were observed for STAT3, suggesting that this protein may represent indirect downstream effectors rather than primary molecular targets of MBF. Collectively, these findings strengthen the hypothesis that HSP90AA1 and EGFR constitute the principal molecular hubs associated with the anticancer activity of MBF.

Although STAT3 emerged as the highest-ranked hub gene in the topological analysis of the PPI network, molecular docking suggested a comparatively weaker interaction with MBF. Nevertheless, given its extensive connectivity within the network and its established role in regulating cell survival, inflammation, metabolic adaptation, and resistance to apoptosis [62,64], STAT3 may still contribute indirectly to the observed biological effects [75]. Thus, rather than acting as a primary molecular target, STAT3 may represent a downstream signaling node affected by MBF-mediated disruption of upstream regulators such as HSP90AA1 and EGFR. Identification of STAT3 as one of the hub proteins points toward modulation of the pro-inflammatory tumor microenvironment since JAK/STAT3 activation could take place through IL-6, which supports cell survival and therapy resistance [9,76]. Interestingly, CIP–fatty acid conjugates significantly decreased IL-6 secretion in cancer cells, potentially inhibiting tumor progression [34]. Given its central position within the network, STAT3 signaling may still contribute to the biological effects of MBF indirectly through downstream modulation of inflammatory and survival-associated pathways.

KEGG pathway enrichment analysis of the overlapping 73 genes further supported these findings by revealing a significant enrichment of pathways critical in oncogenic cascades, including pathways in cancer, FoxO signaling, HIF-1 signaling, IL-17 signaling, chemokine signaling, PD-L1/PD-1 checkpoint signaling, and cAMP signaling pathways. These pathways fundamentally regulate cell proliferation, stress adaptation, and apoptotic commitment [77,78,79,80,81]. Specifically, the enrichment of FoxO signaling pathway could provide a direct mechanistic link to the induction of cellular senescence and suppressed growth observed in our findings. FoxO transcriptional factors have pivotal roles in cancer biology, acting as tumor suppressors that trigger cell-cycle arrest and inhibition of proliferation [82,83,84]. A key downstream target of this pathway is *CDKN1A* (p21), a potent cyclin-dependent kinase inhibitor that mediates G1 arrest and senescence [78,85]. The significant upregulation of *CDKN1A* in both cells following MBF treatment is consistent with the KEGG enrichment analysis. Moreover, this transcriptional change was accompanied by the downregulation of key proliferation-associated genes, including *CCND1* and *CCNE1* in MCF-7 cells and *MKI67* in both cell lines, further supporting the anti-proliferative effects of MBF. Furthermore, the enrichment of HIF-1 and cAMP signaling pathways aligns with the metabolic alterations observed in MBF-treated cells. This interpretation is further supported by subtype-specific metabolic remodeling involving glutathione metabolism, nucleotide metabolism, polyamine metabolism, coenzyme A biosynthesis, and distinct alterations in detoxification- and lipid-associated metabolites identified through untargeted metabolomic profiling, consistent with suppressed proliferation and cellular stress responses.

Taken together, the integration of phenotypic, molecular, metabolomic, network pharmacology, molecular docking, and molecular dynamics analyses suggests that MBF exerts its anticancer activity through a multi-target mechanism involving disruption of putative HSP90AA1–EGFR-associated oncogenic networks, impairment of metabolic homeostasis, and activation of oxidative stress-mediated cell death programs. The convergence of experimental and computational findings highlights MBF as a promising repurposing candidate capable of simultaneously targeting proliferative, metabolic, and survival pathways in breast cancer cells.

Several limitations of the present study should be acknowledged. First, although the network pharmacology, enrichment, and molecular docking analyses identified several biologically relevant targets, including EGFR, STAT3, HSP90AA1, and MMP9, these interactions remain computational predictions and were not experimentally validated. Therefore, the direct molecular targets of MBF and their contribution to the observed phenotypic effects require further investigation using protein expression analyses, pathway-specific inhibition studies, and genetic gain- or loss-of-function approaches. Second, while the present study provides comprehensive evidence of MBF-mediated growth inhibition, apoptosis, senescence, metabolic, and transcriptional alterations in BC cells, the precise causal relationships between these events remain to be fully elucidated. Whether oxidative stress and metabolic perturbations represent primary mechanisms of action or secondary consequences of upstream signaling alterations warrants additional investigation. In particular, rescue experiments using antioxidants such as N-acetylcysteine will be required to determine whether oxidative stress directly mediates MBF-induced cytotoxicity. Third, the current findings were generated using in vitro BC models and should therefore be validated in vivo; employing animal models, pharmacokinetic assessments, and toxicity evaluations will be necessary to determine the translational relevance and therapeutic feasibility of MBF in BC. Furthermore, although HEK-293 cells provided an initial non-malignant human comparator for evaluating differential cytotoxicity, they do not represent normal mammary epithelium. Future studies employing immortalized non-tumorigenic breast epithelial cell lines, such as MCF-10A or MCF-12A, will be necessary to more rigorously assess tissue-specific selectivity of MBF.

## 4. Materials and Methods

### 4.1. Cell Culture Maintenance & Drug Preparation

Human epithelial breast cancer cell lines MCF-7 (ATCC, Manassas, VA, USA, #HTB-22) and MDA-MB-231 (ATCC, Manassas, VA, USA, #CRM-HTB-26) were used from our own stocks. All cells were maintained in Dulbecco’s Modified Eagle Medium (Sigma-Aldrich, St. Louis, MO, USA, #D6429) supplemented with 10% Fetal Bovine Serum (Thermo Fisher Scientific, Waltham, MA, USA, #A4736401), 100 IU/mL penicillin, and 100 mg/mL streptomycin (Thermo Fisher Scientific, Waltham, MA, USA, #15140122). The cells were cultured at 37 °C in a humidified atmosphere supplemented with 5% CO_2_. Passaging was carried out upon attaining approximately 80% confluence. Cell proliferation was routinely assessed under an inverted microscope (SOPTOP ICX41, Ningbo, China). Stock solutions of MBF (MedChem Express, Monmouth Junction, NJ, USA, #HY-B0126) were prepared at a concentration of 5 mM in dimethyl sulfoxide (DMSO; PAN-Biotech, Aidenbach, Germany, #P60-36720100). Working concentrations were generated by serial dilution of the stock solution directly into the culture medium. All subsequent assays were performed in three independent replicates.

### 4.2. Evaluation of Cell Viability

Cell viability was evaluated employing the CellTiter 96^®^ Non-Radioactive Cell Proliferation Assay Kit (Promega, Madison, WI, USA, #G4000). The MCF-7 and MDA-MB-231 breast cancer cells were seeded into 96-well plates at a density of 1 × 10^4^ cells in 100 μL of culture medium and incubated for 24 h to allow adherence. Cells were then treated with MBF at a range of 0.001–0.5 mM for 24 h.

Negative control cells received culture medium supplemented with DMSO at a concentration equivalent to that used as the vehicle for the tested compounds, while positive controls were exposed to 10% DMSO. The assay dye was added to each well, followed by a 4 h incubation; the reaction was subsequently stopped with the solubilization/stop mix. Absorbance was measured at 570 nm (MultiscanGO, Thermofisher Scientific, Waltham, MA, USA). The protocol was replicated using a seeding density of 5 × 10^3^ cells/well for a 48 h treatment duration. IC_50_ values were calculated by nonlinear regression of dose–response curves via the “drc” package in R (v4.4.1), as previously described [86].

### 4.3. Assessing Crystal Violet-Based Colony Outgrowth of the Cells

To assess the colony-forming capacity of cells after drug treatment, a clonogenic assay was performed. MCF-7 and MDA-MB-231 cells were seeded in 2 × 10^4^ cells per well in 96-well plates with 100 μL of culture medium. MBF concentrations were chosen based on IC_50_ values obtained from the cell viability assay for both cell lines (1/2 IC_50_, IC_50_, and 2× IC_50_). Treated cells were continuously exposed to MBF and control cells received vehicle culture medium, and the medium in all groups was refreshed every 48 h until a minimum of 80% confluency was reached. Colonies were then fixed in 100% methanol (Sigma Aldrich, St. Louis, MO, USA, #24229) for 20 min at room temperature (RT), stained with 0.2% Crystal Violet (Sigma, St. Louis, MO, USA, #C0775) for 15 min, and rinsed twice with ddH_2_O to remove excess stain. Plates were air-dried, imaged via bright-field microscopy (SOPTOP ICX41, Ningbo, China), and quantified for colony number and intensity using the “ColonyArea” plugin in ImageJ (version 1.53e).

### 4.4. Determining Tumorigenicity of the Cells

To evaluate the anchorage-independent growth—a hallmark of tumorigenicity—in MCF-7 and MDA-MB-231 breast cancer cells, a soft agar assay was conducted. A 5% agar (Sigma, St. Louis, MO, USA, #A6686) stock solution was prepared, sterilized by autoclaving, and diluted in culture medium to yield working concentrations. Per well, 800 µL of 0.5% agar formed the bottom layer, followed by 800 µL of 0.3% agar as the intermediate layer; the top layer comprised vehicle medium alone (control group) or supplemented with MBF at the 24 h IC_50_ concentration, refreshed periodically during incubation. Colonies were imaged via bright-field microscopy (SOPTOP ICX41, Ningbo, China), with number and size quantified using ImageJ’s “ParticleSizer” plugin as described earlier [87].

### 4.5. Measuring the Migration Capacity of the Cells

To evaluate the migration capacity of MCF-7 and MDA-MB-231 cells following MBF treatment, a wound healing (scratch) assay was conducted. Cells were seeded into 24-well plates containing 1 mL of culture medium and 50,000 cells per well and incubated for 24 h to achieve confluence. The medium was subsequently replaced with MBF-containing medium at the 24 h IC_50_ concentration determined for each cell line or with vehicle medium alone. A uniform cell-free wound was then created using a 200 µL pipette tip. Bright-field images were captured at 0, 18, and 24 h using an inverted microscope (SOPTOP ICX41, Ningbo, China). Wound closure was quantified with the “MRI Wound Healing Tool” plugin in ImageJ software (version 1.53e).

### 4.6. Assessing Apoptosis Rate of the Cells

To assess whether MBF treatment could induce apoptosis, the Alexa Fluor^®^ 488 Annexin V/Dead Cell Apoptosis Kit (Thermo Fisher Scientific, Waltham, MA, USA, #V13242) was employed. MCF-7 and MDA-MB-231 breast cancer cells were seeded into 12-well plates (100,000 cells/well in 1 mL of culture medium) and incubated for 24 h. The medium was subsequently replaced with either MBF-supplemented medium (at the 24 h IC_50_ concentration specific to each cell line) or vehicle medium alone, followed by an additional 24 h incubation. Cells were then harvested, washed with ice-cold phosphate-buffered saline (PBS, Gibco, New York, NY, USA, #18912014), and resuspended in 100 μL of 1x Annexin V-binding buffer. FITC-conjugated Annexin V and 7AAD working solutions were added, with samples incubated for 15 min at RT. After supplementation with 400 μL of 1x Annexin V-binding buffer, apoptotic populations were quantified via flow cytometry (BD Accuri™ C6 Plus, San Jose, CA, USA).

### 4.7. Evaluation of Cell Proliferation

Cell proliferation was evaluated following MBF treatment using the Click-iT™ EdU Cell Proliferation Assay Kit (Thermo Fisher Scientific, Waltham, MA, USA, C10337). MCF-7 and MDA-MB-231 cells were seeded into 96-well plates at a density of 1 × 10^4^ in 100 μL medium and allowed to adhere. Then, the groups were formed by treating the cells with MBF or vehicle medium for 24 h. A quantity of 20 mM working solution of EdU was prepared according to manufacturer instructions and 50 μL of EdU solution was added to each well, followed by a 2 h incubation. Cells were fixed with 100% methanol, washed with PBS containing bovine serum albumin, and permeabilized with a saponin-based reagent. The Click-iT^®^ reaction cocktail, prepared according to the manufacturer’s protocol, was applied for EdU detection. Following incubation for 30 min at RT, cells were counterstained with DAPI dye, imaged via fluorescence microscopy (Nikon Eclipse Ts2, Japan, Tokyo), and quantified for EdU-positive and DAPI-positive cells using ImageJ software (version 1.53e).

### 4.8. Analysis of ΔΨm

The mitochondrial membrane potential was evaluated using the JC-1 (5,5’,6,6’-tetrachloro-1,1’,3,3’-tetraethylbenzimidazolylcarbocyanine iodide) fluorescent probe (Thermofisher Scientific, Waltham, MA, USA, # T3168) to determine the impact of MBF on the ΔΨm of MCF-7 and MDA-MB-231 cells. Cells were seeded in 6-well plates at a density of 3 × 10^5^ cells per well in 2 mL medium and allowed to adhere for 24 h. Following adherence, the cells were treated with MBF at the calculated IC_50_ concentrations for 24 h. After the incubation period, cells were harvested by trypsinization, centrifuged, and washed twice with cold PBS. The resulting cell pellets were resuspended in a JC-1 working solution (5 µM) and incubated at 37 °C in the dark for 30 min. Following incubation, cells were washed to remove excess dye and resuspended in 0.5 mL of assay buffer for flow cytometric analysis. Data acquisition was performed using a flow cytometer equipped with a 488 nm excitation laser (BD Accuri™ C6 Plus).

### 4.9. Analysis of Intracellular ROS Production

MCF-7 and MDA-MB-231 cells were seeded in 6-well plates at a density of 3 × 10^5^ cells/well in 2 mL medium and treated with MBF at 24 h IC_50_ concentrations for 24 h. Following treatment, cells were harvested, washed with PBS, and incubated with 10 µM DCFH-DA (Sigma Aldrich, USA, St. Louis #D6883) in serum-free medium at 37 °C for 30 min in the dark. After incubation, the cells were washed twice with PBS to remove extracellular probe and resuspended in 0.5 mL of PBS for immediate analysis. Fluorescence intensity was measured using a flow cytometer with an excitation wavelength of 488 nm and an emission wavelength of 530 nm (BD Accuri™ C6 Plus, San Jose, CA, USA). A minimum of 10,000 events were acquired per sample, and the results were expressed as the mean fluorescence intensity relative to the control groups.

### 4.10. SA-ß-Gal Staining

To evaluate MBF-induced cellular senescence, SA-ß-gal activity was detected using the Senescence-associated-ß-Galactosidase Staining Kit (Cell Signaling Technology, Danvers, MA, USA, #9860) according to the manufacturer’s instructions. Cells were seeded in 24-well plates and treated with indicated concentrations of MBF. Following the treatment period, the culture medium was aspirated, and the cells were washed once with PBS. The cells were then fixed using the 1× Fixative Solution provided in the kit for 15 min RT. After two subsequent washes with PBS, cells were incubated overnight at 37 °C in a dry incubator with the freshly prepared SA-ß-gal Staining Solution adjusted to pH 6.0. The development of the blue-stained senescent cells was observed under a light microscope (SOPTOP ICX41, Ningbo, China). The percentage of senescent cells was quantified by counting at least 200 cells in five random fields per well and analyzed using ImageJ (version 1.53e).

### 4.11. Evaluating Gene Expression Profile of the Cells

To elucidate the molecular mechanisms underlying the cellular effects induced by MBF, quantitative real-time polymerase chain reaction (qRT-PCR) was performed on a panel of proto-oncogenes and tumor suppressor genes, as previously described [87]. MCF-7 and MDA-MB-231 breast cancer cells were seeded in 6-well plates with 2 mL of culture medium at 3 × 10^5^ cell/well and incubated for 24 h. The medium was then replaced with either vehicle medium or MBF-containing medium, followed by an additional 24 h incubation. Total RNA was extracted using a previously described protocol [88], reverse-transcribed into cDNA, and analyzed by qRT-PCR with gene-specific primers. GAPDH served as the housekeeping gene for normalization, and relative gene expression was quantified using the 2^−ΔΔCt^ method.

### 4.12. Metabolome Profiling

To investigate the metabolic alterations elicited by MBF, untargeted metabolomics profiling was conducted as previously described [88]. In brief, breast cancer cells were seeded in 75 cm^2^ flasks and incubated for 24 h. The culture medium was subsequently replaced with either vehicle medium or MBF-supplemented medium at the respective 24 h IC_50_ concentrations for each cell line, followed by an additional 24 h incubation. Cells were then detached via trypsinization, transferred to 5 mL centrifuge tubes, and pelleted by centrifugation at 1200 rpm for 5 min. Cell pellets were rinsed three times with ice-cold PBS, resuspended in PBS at a 9:1 (*v*/*v*) ratio, and repelleted. Subsequently, 200 µL of extraction solvent was added, and samples were vigorously vortexed. Cell lysis was achieved through five sonication cycles, with samples maintained on ice between cycles. For protein precipitation, extracts were incubated at −20 °C for 1 h, followed by centrifugation at maximum speed for 15 min at 4 °C. The supernatants were collected on a clean plate, and the solvent was evaporated under a nitrogen stream. Dried residues were reconstituted in 50 µL of water, transferred to autosampler vials, and subjected to ultra-high-performance liquid chromatography coupled with mass spectrometry analysis. Chromatographic parameters included a column temperature of 45 °C, sample tray temperature of 8 °C, and injection volume of 1 µL. Mobile phase A comprised ultrapure water with 0.1% formic acid, and mobile phase B consisted of methanol with 0.1% formic acid, delivered via a 30 min gradient at 0.3 mL/min. Electrospray ionization was performed in both positive and negative modes across separate runs, employing MSE data acquisition to capture precursor and fragment ion spectra. Raw LC-MS data underwent peak detection, alignment, and normalization using Compound Discoverer 3.3 SP1 software. Metabolite annotation relied on ChemSpider, Human Metabolome Database, mzCloud, and mzVault databases. Significantly altered metabolites were identified using a fold-change threshold >1.5 and *p* < 0.05. Pathway enrichment and topological analysis were executed with MetaboAnalyst 6.0 [89] and the Kyoto Encyclopedia of Genes and Genomes (KEGG) database. Exogenous metabolites were verified to reduce false positives, acknowledging potential inclusion of structurally similar isomers from database matching. Differential metabolites were depicted via EnhancedVolcano plots in R.

### 4.13. Network Pharmacology and PPI Analysis

To identify the potential molecular targets of MBF in BC, a network pharmacology approach was employed. BC-associated genes were retrieved from the GeneCards database [90] using the keyword “breast cancer”, and only genes with a relevance score >10 were retained for further analysis. The canonical SMILES structure of MBF was obtained from the PubChem database [91] and subsequently submitted to the SwissTargetPrediction platform [92] to predict its putative molecular targets. The overlap between MBF-associated targets and BC-related genes was identified using the ggvenn package in R, and the intersecting genes were considered potential therapeutic targets of MBF in BC.

To investigate the interactions among the overlapping targets, a PPI network was constructed using the STRING database (version 12.0) [93], with *Homo sapiens* selected as the reference organism as described earlier [94]. The resulting interaction network was imported into Cytoscape software (v3.9.1) [95] for visualization and topological analysis.

Hub genes within the PPI network were identified using the CytoHubba plugin [96] in Cytoscape. Two complementary topological algorithms, Degree and MCC, were applied to rank nodes according to their relative importance within the network. The top 10 genes identified by each algorithm were extracted, and common hub genes were selected for downstream computational analyses.

To further explore the biological pathways potentially associated with the identified targets, Kyoto Encyclopedia of Genes and Genomes (KEGG) pathway enrichment analysis was performed using the clusterProfiler package [97] in R. Pathways with an adjusted *p*-value < 0.05 were considered significantly enriched. The enrichment results were visualized using dot plots generated with the enrichplot package to illustrate the most significantly enriched signaling pathways associated with the potential anticancer activity of MBF in BC.

### 4.14. Molecular Docking Studies

The three-dimensional structures of MBF (CID: 60651), osimertinib (CID: 71496458), SI109 (CID: 404647587), and 4-chloranyl-7-[(4-methoxy-3,5-dimethyl-pyridin-2-Yl)methyl]-5-(phenylmethyl)pyrrolo[2,3-D]pyrimidin-2-amine (HSP90A inhibitor) (CID: 131704417) were retrieved from the PubChem database. Prior to molecular docking, all ligand structures were energy-minimized using Open Babel integrated in PyRx 0.8. The crystal structures of the target proteins complexed with their respective inhibitors, namely HSP90A (PDB ID: 5H22), EGFR (PDB ID: 9BY4), and STAT3 (PDB ID: 6NUQ), were obtained from the RCSB Protein Data Bank [98], while the co-crystallized ligands were employed as reference inhibitors. Protein preparation was conducted using UCSF Chimera [99] by removing bound ligands and other non-essential molecules to ensure compatibility for docking analysis. Molecular docking analyses were performed using AutoDock Vina (v1.2.x) implemented in PyRx 0.8 targeting the ligand-binding domains of HSP90A, EGFR, and STAT3. Furthermore, the stability of the protein–ligand complexes was evaluated independently using the CABS-flex 3.0 server [100] with default settings. For each protein target, multiple binding poses were generated and ranked according to their predicted binding affinities. The reported RMSD lower-bound and upper-bound values correspond to the internal AutoDock Vina output, which measures the deviation of each predicted pose relative to the top-ranked docking conformation (Mode 1). Therefore, the first-ranked docking pose has RMSD values of 0.000 Å by definition, and these values should not be interpreted as the RMSD relative to the experimentally determined co-crystallized ligand. The final docking pose selected for each ligand–protein complex was determined based on a comprehensive evaluation of the docking results. The selection criteria included the predicted binding affinity, localization within the reported or predicted active binding site, the formation of interactions with key amino acid residues known to be involved in ligand recognition or catalytic activity, and the overall plausibility of the binding orientation. Docking poses were ranked according to their predicted binding free energy, and the most favorable conformations were analyzed for their key molecular interactions. Visualization of the docked complexes was carried out using UCSF Chimera and ProteinsPlus. Residue flexibility was assessed through root mean square fluctuation (RMSF) analysis to compare the dynamic behavior of receptors in the ligand-bound complexes with those of the reference inhibitor complexes.

### 4.15. Statistical Analysis

All experimental data are expressed as the mean ± standard deviation. Normality of data distribution was evaluated using the Shapiro–Wilk test. Statistical comparisons for the cell viability assay and relative gene expression analysis were performed using one-way analysis of variance (ANOVA) followed by Tukey’s post hoc test. For all other assays, significance was determined with the Student’s *t*-test. A *p* < 0.05 was considered statistically significant. All statistical analyses and data visualizations were conducted using the “rstatix” package in R software (version 4.6.1).

## 5. Conclusions

Taken together, the integration of phenotypic, molecular, metabolomic, network pharmacology, molecular docking, and RMSF-based analyses suggests that MBF exerts anti-cancer activity in breast cancer cells through the coordinated modulation of proliferation, apoptosis, oxidative stress, mitochondrial function, and metabolic homeostasis. In particular, MBF treatment was associated with reduced cell viability, impaired clonogenic and anchorage-independent growth, decreased DNA synthesis, increased ROS accumulation, mitochondrial membrane depolarization, apoptosis, and a senescence-like phenotype in both MCF-7 and MDA-MB-231 cells. Metabolomic profiling further indicated broad remodeling of growth-supporting metabolic pathways, including alterations in polyamine, nucleotide, glutathione, energy-associated, and lipid-related metabolism. In parallel, network-pharmacology and molecular-docking analyses predicted EGFR and HSP90AA1 as candidate molecular nodes that may be involved in MBF-associated anti-cancer activity; however, these predicted interactions require direct experimental validation. Overall, the present findings provide initial in vitro evidence supporting the anti-neoplastic potential of MBF in breast cancer cell models and suggest that this compound may represent a candidate for further preclinical investigation. Nevertheless, the current results should be interpreted within the limitations of an in vitro study. Future work should include non-tumorigenic breast epithelial controls, protein-level validation of predicted molecular targets, pathway-specific rescue or inhibition experiments, pharmacokinetic and toxicity assessments, and in vivo breast cancer models to determine the selectivity, mechanistic relevance, and translational feasibility of MBF as a repositionable anti-cancer candidate.

## Figures and Tables

**Table 1:** Half-maximal inhibitory concentration (IC_50_) values of MBF in BC and HEK-293 cell lines following 24 and 48 h treatment. HEK-293 IC_50_ values are reported as >500 µM, the highest concentration tested; the exact IC_50_ could not be determined within the tested concentration range.

Cell Line	IC_50_ (24 h, μM)	IC_50_ (48 h, μM)
MCF-7	377	93
MDA-MB-231	84	18
HEK-293	>500	>500

**Table 2:** Relative expression of genes associated with cell cycle regulation, apoptosis, proliferation, and migration in MBF-treated BC cells. ↓ indicates downregulation while ↑ indicates upregulation. ns; non-significant. * *p*-value < 0.05. ** *p*-value < 0.01. *** *p*-value < 0.001. ns; non-significant. Data are presented as the mean ± SD of three independent biological replicates.

Gene	MCF-7 Cells (Fold Change)	MDA-MB-231 Cells (Fold Change)
*ANKRD1*	↓ 10.29 ***	↓ 9.35 ***
*BIRC5*	↓ 2.00 ***	ns
*BCL2*	ns	↓ 2.23 **
*CCND1*	↓ 2.66 ***	ns
*CCNE1*	↓ 3.30 ***	ns
*CDK6*	↓ 2.80 ***	ns
*CDKN1A*	↑ 4.00 ***	↑ 3.71 **
*CDKN1B*	ns	ns
*CSF2*	ns	↓ 1.87 *
*EDN1*	ns	↓ 3.89 **
*MKI67*	↓ 1.76 **	↓ 2.96 *
*MMP9*	↓ 4.54 ***	ns
*PUMA*	↑ 2.00 **	ns

**Table 3:** Comparative molecular docking analysis of MBF and co-crystallized reference inhibitors with EGFR, STAT3, and HSP90A.

Target	Compound	Binding Affinity (kcal/mol)	RMSD Lower (Å)	RMSD Upper (Å)	Hydrogen Bonds	Hydrophobic Interactions
EGFR	Inhibitor (osimertinib)	−7.6	0.000	0.000	**Lys745A**	Phe723A; **Val726A;** Leu718A
EGFR	MBF	−7.2	2.822	4.018	Thr790A; **Lys745A**	**Val726A**
STAT3	Inhibitor (SI109)	−7.3	0.000	0.000	Tyr657A; Gln644A; Glu638A; Ser636A; Glu612A; Ser613A; Ser611A	Pro639A; Val637A; Glu638A; Ser636A
STAT3	MBF	−5.4	3.481	5.715	Ala578A	Ser649A
HSP90A	Inhibitor	−10.1	0.000	0.000	**Asp93A;** Phe138A	**Leu107A**; **Met98A**; Phe138A
HSP90A	MBF	−8.4	0.000	0.000	**Asp93A;** Ser52A	**Met98A**; **Leu107A**

Amino acid residues involved in interactions at the same binding positions as those of the corresponding co-crystallized reference inhibitor are highlighted in bold. MBF, marbofloxacin; EGFR, epidermal growth factor receptor; STAT3, signal transducer and activator of transcription 3; HSP90A, heat shock protein 90 alpha family class A member 1; RMSD, root mean square deviation.

## Data Availability

The original contributions presented in the study are included in the article, further inquiries can be directed to the corresponding authors.

## Abbreviations

The following abbreviations are used in this manuscript:

BC	Breast cancer
MBF	Marbofloxacin
FQ	Fluoroquinolone
TNBC	Triple-negative breast cancer
ER	Estrogen receptor
IC_50_	Half-maximal inhibitory concentration
MMP	Matrix metalloproteinase
ΔΨm	Mitochondrial membrane potential
ROS	Reactive oxygen species
EdU	5-Ethynyl-2′-deoxyuridine
SA-ß-gal	Senescence-associated ß-galactosidase
qRT-PCR	Quantitative real time polymerase chain reaction
PPI	Protein–protein interaction
STRING	Search tool for the retrieval of interaction genes/proteins
MCC	Maximal clique centrality
KEGG	Kyoto Encyclopedia of Genes and Genomes
EGFR	Epidermal growth factor receptor
STAT3	Signal transducer and activator of transcription 3
HSP90AA1	Heat shock protein 90 alpha family class A member 1
JAK2	Janus kinase 2
MAPK	Mitogen-activated protein kinase
GSH	Glutathione
UDP	Uridine diphosphate
RMSD	Root mean square deviation
